# Helium Cold Atmospheric Plasma Causes Morphological and Biochemical Alterations in *Candida albicans* Cells

**DOI:** 10.3390/molecules28237919

**Published:** 2023-12-03

**Authors:** Sabrina de Moura Rovetta-Nogueira, Aline Chiodi Borges, Maurício de Oliveira Filho, Thalita Mayumi Castaldelli Nishime, Luis Rogerio de Oliveira Hein, Konstantin Georgiev Kostov, Cristiane Yumi Koga-Ito

**Affiliations:** 1 Oral Biopathology Graduate Program, Department of Environment Engineering, São José dos Campos Institute of Science & Technology, São Paulo State University (UNESP), São José dos Campos 12247-016, SP, Brazil; sabrinam_sjc@yahoo.com.br (S.d.M.R.-N.); aline_chiodi@hotmail.com (A.C.B.); 2 Department of Materials and Technology, Guaratinguetá Faculty of Engineering and Sciences, São Paulo State University (UNESP), Guaratinguetá 12516-410, SP, Brazil; mauricio.oliveira@unesp.br (M.d.O.F.); rogerio.hein@unesp.br (L.R.d.O.H.); 3 Leibniz Institute for Plasma Science and Technology, 17489 Greifswald, Germany; thalita.nishime@inp-greifswald.de; 4 Department of Physics, Guaratinguetá Faculty of Engineering, São Paulo State University (UNESP), Guaratinguetá 12516-410, SP, Brazil; konstantin.kostov@unesp.br

**Keywords:** *Candida albicans*, cold atmospheric plasma, AFM, FTIR, Caspofungin, chitin, glucans

## Abstract

(1) Background: Previous studies reported the promising inhibitory effect of cold atmospheric plasma (CAP) on *Candida albicans*. However, the exact mechanisms of CAP’s action on the fungal cell are still poorly understood. This study aims to elucidate the CAP effect on *C. albicans* cell wall, by evaluating the alterations on its structure and biochemical composition; (2) Methods: *C. albicans* cells treated with Helium-CAP were analyzed by atomic force microscopy (AFM) and Fourier transform infrared spectroscopy (FTIR) in order to detect morphological, topographic and biochemical changes in the fungal cell wall. Cells treated with caspofungin were also analyzed for comparative purposes; (3) Results: Expressive morphological and topographic changes, such as increased roughness and shape modification, were observed in the cells after CAP exposure. The alterations detected were similar to those observed after the treatment with caspofungin. The main biochemical changes occurred in polysaccharides content, and an overall decrease in glucans and an increase in chitin synthesis were detected; (4) Conclusions: Helium-CAP caused morphological and topographic alterations in *C. albicans* cells and affected the cell wall polysaccharide content.

## 1. Introduction

*Candida albicans* is an opportunistic fungal pathogen that can cause both superficial and systemic infections and is a causative agent of hospital acquired infections [1,2,3,4,5,6,7]. Currently, these infections are considered a serious public health problem, due to the high rates of morbidity [1,8] and mortality [2,3,5].

There are important challenges for the treatment of fungal infections, such as the high toxicity of the available antifungals [9] and the limited number of drugs that are suitable for clinical use [1,5,9,10,11,12,13,14,15,16]. In fact, the discussion on growing antifungal resistance of *Candida albicans* has been intense in the last decade [17], but it seems that the global efforts have not been enough to prevent and control the occurrence of infection. The discovery of new antifungals that can be effectively used in clinical practice is a concern and a challenge [8,9].

Plasmas are partially or totally ionized gases that are abundant with charged particles, electromagnetic radiation, and diverse reactive radicals and excited species [18]. These plasma components and their properties have been particularly useful in several fields, such as agriculture, medicine, dentistry, and environmental areas [18]. Biological effects of cold atmospheric plasmas (CAP) have been closely related to the presence of oxygen and nitrogen species (RONS), ions and UV radiation, which can cause damage to the microbial cell structures, such as the plasmatic membrane [19,20], cell wall [20,21], proteins [19,20,21] and DNA [19,20].

Previous studies reported promising inhibitory activity of CAP on *C. albicans*, both in planktonic and biofilm forms [22,23,24,25,26,27,28,29,30,31,32]. In vivo studies corroborate these findings, and a significant reduction in fungal tissue invasion was detected [26,31]. However, the investigations on the CAP effects, specifically on the cell components or the virulence factor of *C. albicans* cells, are scarce. A reduction of 40–91% in ergosterol biosynthesis was observed by Rahimi-Verki et al. [33]. CAP also inhibited the fungal filamentation by almost 40 times and reduced the capacity of adhesion to mammal cells [32]. Previous works [32,33] reported reduction in the production of exoenzymes, such as phospholipase and proteinase. 

The fungal cell wall has been considered an excellent target for antifungal therapy due to its differential composition when compared to human cells [7]. The fungal cell wall is one of the main structures responsible for maintaining morphology and protecting the cell against chemical and physical damage [34]. It plays a central role in the fungus–host interaction, due to the polysaccharides, which interact with the receptors of the immune system [34,35,36,37,38]. *C. albicans* cell wall contains 80–90% carbohydrate [34], and the main biomolecules are glucans, mannoproteins and chitins. Each of these components is responsible for a specific biological activity that ranges from fungal maintenance and survival to pathogenicity mechanisms and the process of recognition by the host’s immune system [34]. 

The major constituents of the cells’ outer layer are the mannose polymers (mannans) that are usually associated with proteins, forming glycoproteins. Proteins are involved in adhesion, cell wall structuring and remodeling [34,35,36,37,38]. The inner layer of the cell wall contains chitin and glucans and confers resistance and cell shape [34,38]. Glucans are very important components that maintain the integrity of the fungal cell wall, and changes in their structure lead to an imbalance of the intracellular osmotic pressure [2,34,39]. Chitin is essential for cell survival due to its significant contribution to the rigidity of the cell wall and its role in cell division [7,40].

Cold atmospheric plasma (CAP) inhibitory effect on *Candida albicans*, both in vitro and in vivo, suggests that the plasma treatment can be a promising therapeutic tool, especially in cases refractory to conventional treatment. However, the effects of CAP on the fungal cell are not fully understood so far. This information can contribute to improve the therapeutic protocols for candidiasis, exploring associations with conventional drugs with similar targets on the fungal cells. Thus, the aim of the present work was to investigate whether *C. albicans* cell wall is affected by CAP action. To achieve this goal, the alterations on the fungal cell wall structure, as well as in its biochemical composition, were assessed.

## 2. Results

### 2.1. Antifungal Activity of CAP on Candida albicans and Determination of the Minimum Inhibitory Concentration (MIC) of Caspofungin

The exposure of *C. albicans* suspensions to He-CAP for 120 s reduced the number of viable cells by 35.5%. The MIC of caspofungin was 0.0625 mg/mL

### 2.2. Morphological and Topographical Evaluation of Candida albicans Cells Exposed to CAP by Atomic Force Microscopy (AFM)

*C. albicans* cells exposed to CAP for 120 s were compared to cells treated with caspofungin (a positive control) and non-exposed cells (a negative control).

**Figure 1:**
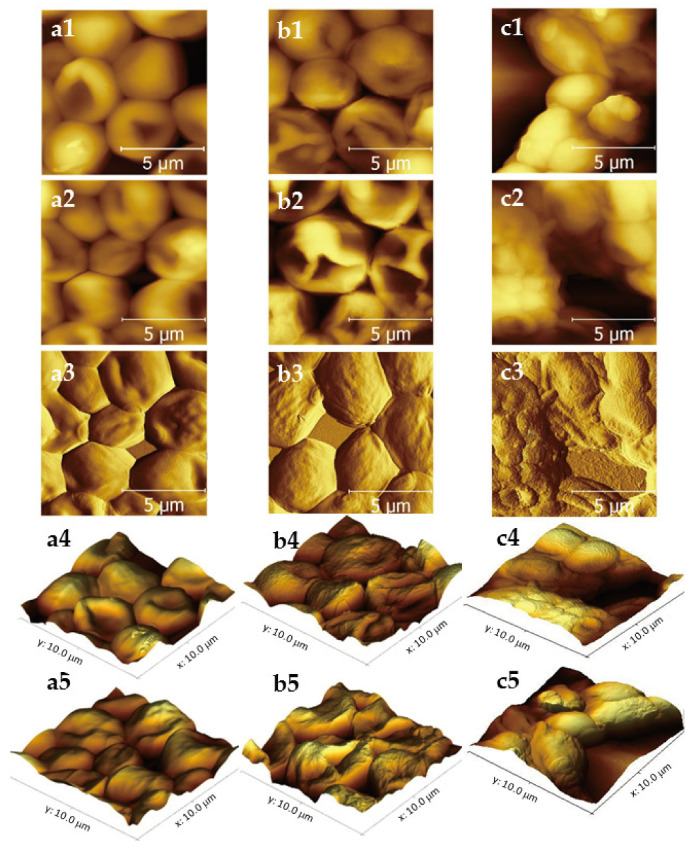
AFM morphological evaluation of *Candida albicans* cells exposed to cold atmospheric plasma ((**b1**,**b2**) in 2D mode; (**b3**) in deflection mode; (**b4**,**b5**) in 3D mode), caspofungin (positive control) ((**c1**,**c2**) in 2D mode; (**c3**) in deflection mode; (**c4**,**c5**) in 3D mode) and control (negative control) ((**a1**,**a2**) in 2D mode; (**a3**) in deflection mode; (**a4**,**a5**) in 3D mode).

AFM micrographs in 2D shown in Figure 1(a1,a2) revealed that the untreated cells exhibited regular morphology with standard characteristics: oval shape and smooth surfaces (Figure 1(a1,a2)). The AFM micrograph in the deflection mode (see Figure 1(a3)), evidenced scars of reproduction and a mild roughness.

After exposure to CAP, significant changes in the cells’ morphology were detected. Micrographs in 2D (Figure 1(b1,b2)) and 3D (Figure 1(b4,b5) showed many cells with significant alterations in their surface topography and with visibly higher roughness. In addition, cells exposed to CAP shrank, showing on average a 13.5% reduction in the cell size in relation to the negative control. Treatment with caspofungin caused an even larger cell size reduction in comparison to the control (35.7%) (Table 1).

As evidenced by Figure 1(c1,c2), *C. albicans* cells exposed to CAP, as well as the ones treated with caspofungin, exhibited various crests and depressions in their surfaces. Micrographs in the deflection mode (Figure 1(c3) also showed cells with wrinkled surfaces and increased roughness.

**Figure 2:**
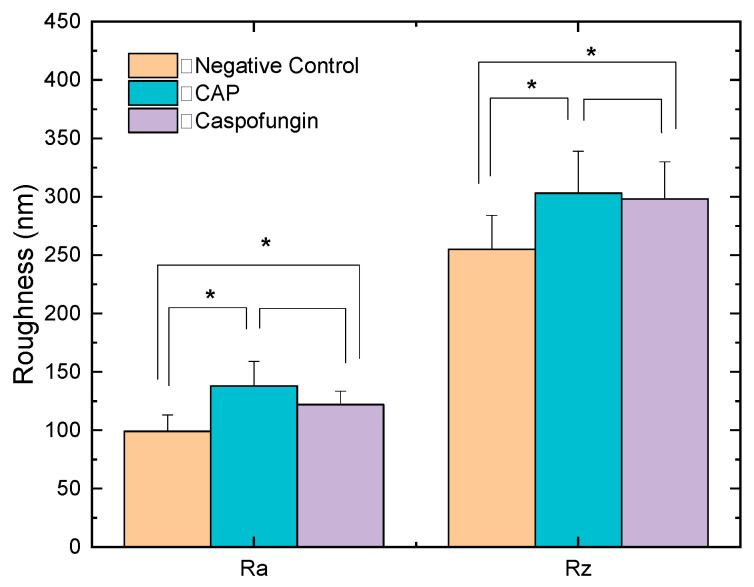
Mean and standard deviation of roughness parameters measurements (arithmetic average, Ra, and peak-to-valley height, Rz) of cells exposed to cold atmospheric plasma (CAP) and caspofungin (positive control) in relation to control (negative control). * *p* < 0.05 (ANOVA and Tukey’s post hoc test).

Figure 2 shows a comparison of the cell’s roughness parameters Ra and Rz (arithmetic average and peak-to-valley height) obtained from the AFM analysis. The ratio between the surface roughness of cells exposed to CAP and the untreated ones increased by 28.3% and 15.8% the Ra and Rz parameters, respectively. Cells treated with caspofungin showed a lower roughness ratio and an increase of 18.8% and 14.4% for Ra and Rz was detected (Figure 2). As can be seen, the changes in cell morphology after CAP exposure were expressive and could also be detected in the 3D micrographs (Figure 1(b4,b5)) and in the deflection mode (Figure 1(b3). After the caspofungin treatment, only mild cell surface irregularities could be noted in the 3D micrographs (Figure 1(c4,c5).

### 2.3. Biochemical Evaluation of Candida albicans Cell Wall Exposed to CAP by Fourier Transform Infrared Spectroscopy (FTIR)

#### 2.3.1. General Comparison

**Figure 3:**
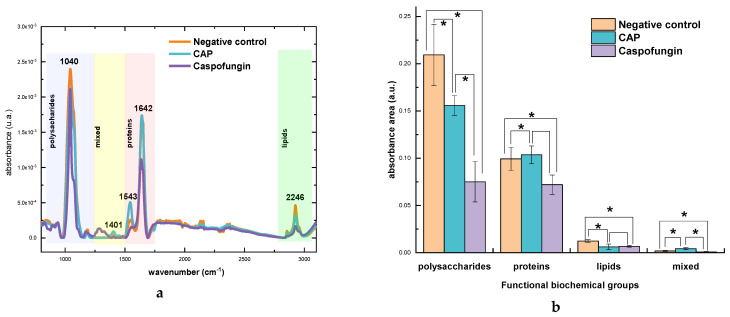
(**a**) Spectra of *Candida albicans* cell wall exposed to cold atmospheric plasma (CAP), caspofungin (positive control) and negative control. Blue represents the absorption region of polysaccharides, yellow represents the mixed region, light pink represents the protein region and green represents the lipid region. (**b**) graph of the main biochemical bands area. * *p* < 0.05.

Infrared spectra exhibited the typical biochemical compounds of the *C. albicans* cell walls, namely polysaccharides (blue), proteins (pink), and lipids (green) [41,42] (Figure 3a). The region in which molecules of polysaccharides and proteins absorb is called mixed region (yellow) [41,42].

The graph in the Figure 3b with information on the band area showed that polysaccharides region is the major contributor to the spectra, followed by the proteins and lipids. Some significant differences in the bands area of different biochemical components of the cell wall could be detected.

#### 2.3.2. Alterations in Polysaccharide Content

**Figure 4:**
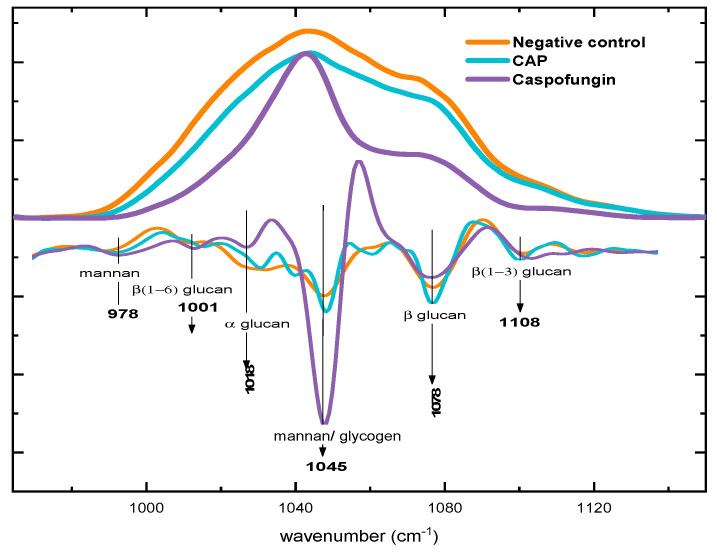
Extended spectra in the polysaccharide region with the second derivatives.

There was a significant reduction in the polysaccharide content after exposure to CAP and caspofungin (Figure 4). After treatment with CAP, there was a 25.6% reduction in band area compared to the negative control group (Figure 3b and Figure 4), while this reduction after treatment with caspofungin was even more expressive (64.2%) (Figure 3b and Figure 4) and (Table 2).

After exposure to CAP and caspofungin, the second derivative of the spectra showed that the intensities of β-glucans bands (1078 and 1108 cm^−1^) [41,42,43] decreased. A reduction in the intensity of glucans bands after exposure to CAP was also observed in the second derivative spectrum (Figure 4).

On the other hand, after exposure to CAP, the intensity of mannans band (978 cm^−1^) and the band assigned to mannan/glycogen (1046 cm^−1^) in the second derivative [41,42,44] were increased when compared to the negative control. Nonetheless, the treatment with caspofungin led to the even higher intensity among those groups (Figure 4).

#### 2.3.3. Alterations in Protein Content

**Figure 5:**
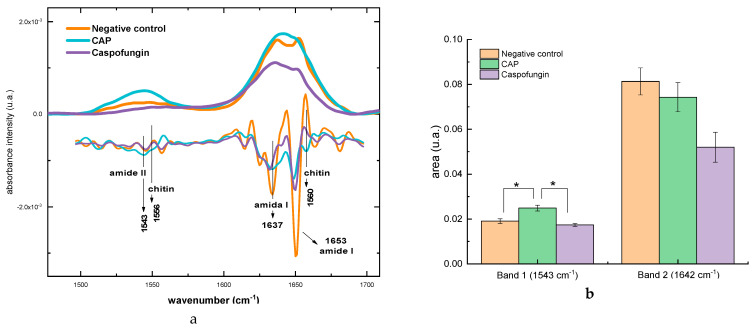
(**a**) Spectra of proteins region with second derivative and (**b**) graph of amide band areas. * *p* < 0.05.

The total area of the protein region, which is the sum of the bands centered in 1540 and 1637 cm^−1^ [44,45], was different after exposure to CAP and caspofungin (Figure 5a) (Table 2). Analyzing each band individually, there was an increase of 23% in the intensity of the band centralized at 1543 cm^−1^ (amide II) [42] in the CAP group in relation to the negative control group. This increase is related to the absorption bands of amide II (1543 cm^−1^) and chitin (1556 cm^−1^), as shown by the second derivative of the spectra (Figure 5a).

The area of the second band, centered at 1642 cm^−1^ (amide I) [42], was compared among the different groups, and a reduction in the absorption intensity of the amide II band for the samples treated with caspofungin was detected (1637 cm^−1^ and 1642 cm^−1^). No statistical difference between the CAP and negative control was detected (Figure 5b). Additionally, a significant difference between the CAP and the caspofungin groups was detected, which suggests that CAP had no effect on these components.

The peak that corresponds to chitin (1560 cm^−1^), exhibited a different pattern, where an increase in intensity in the CAP group in relation to the other groups was noticed. 

The analysis of the second derivative of the spectra showed that the exposure to CAP enhanced the intensity of the chitin peaks, which could have influenced the increase in the general intensity of the protein bands.

#### 2.3.4. Alterations in the Mixed Region

**Figure 6:**
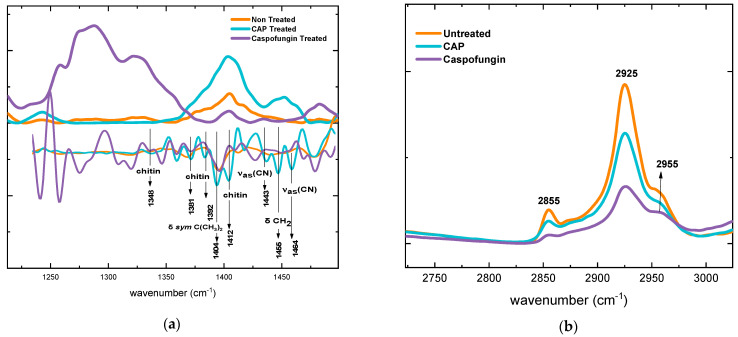
(**a**) Spectra in the mixed region with the spectra second derivative; and (**b**) spectral region of lipids.

The analysis of the mixed region of the spectra showed that, after exposure to CAP, chitin synthesis increased significantly in relation to the negative control (Figure 6a). In fact, the higher presence of the polysaccharide in the CAP group directly influenced the increase in the area of the mixed region (1340–1450 cm^−1^), giving a ratio of 54% in comparison to the negative control group (Table 2). An increase of 63% in relation to the negative control was detected after the cells’ exposure to caspofungin. 

The second derivative of the spectra in the mixed region revealed bands related to the chitin (1348 cm^−1^, 1381 cm^−1^, 1392 cm^−1^ and 1412 cm^−1^) [45] with greater intensities in the CAP and caspofungin groups, when compared to the negative control (Figure 6a). The bands at 1404 cm^−1^ (δ sym C(CH_3_)_2_) are related to proteins, carbohydrates, and lipids, whereas the band at 1455 cm^−1^ (δ CH_2_) corresponds to lipids and proteins [46]. These bands’ intensities were higher after treatment with CAP when compared to the caspofungin and the negative control. The same behavior was observed for the bands at 1412 cm^−1^ (C-O-H), 1443 cm^−1^ and 1464 cm^−1^ (ν_as_ (CN)) that are related to protein vibrational modes [47].

#### 2.3.5. Alterations in the Lipid Content

The analysis of the lipid absorption region showed that the characteristic bands at 2853 cm^−1^ (lipids CH_2_) and 2926 cm^−1^ (lipids CH_2_) [42,43] had a reduction in the absorption intensity after exposure to CAP and caspofungin (Figure 6b). For the CAP-treated cells, a reduction of 47.1% in relation to the negative control was calculated. There were no significant differences between the groups exposed to CAP and caspofungin (*p* > 0.05).

## 3. Materials and Methods

### 3.1. Plasma Source

The atmospheric pressure plasma source used in this work was reported in a previous work [23]. It consisted of a dielectric barrier discharge (DBD) reactor connected to a 1.0-m-long flexible plastic tube (2.5 mm inner diameter and 3.3 mm outer diameter). A thin Cu wire at floating potential was inserted inside the plastic tube. The floating wire penetrated a few millimeters inside the DBD reactor and terminated a few millimeters before the plastic tube end. The DBD reactor consisted of a cylindrical dielectric enclosure, inside which was centered a pin electrode encapsulated inside a quartz tube with a closed bottom end. The pin electrode was connected to a commercial high voltage power supply, Minipuls4 (GBS Elektronik GmbH, Herborn, Germany), which could provide a voltage signal up to 40 kVp-p within a frequency range between 20 and 40 kHz. When a working gas (He in our case) was flushed into the reactor and the high voltage was on, discharge was ignited in the quartz tube vicinity. Therefore, in this condition (with plasma inside the DBD reactor), the floating metal wire acquired a high potential, which in turn generated a small plasma plume at the distal end of the plastic tube. This remote plasma jet is cold and has already been applied for different applications. The parameters used in this work are the same as those used formerly in [21] (frequency of 32 kHz and voltage amplitude of 13.0 kV). To prevent excessive heating of the target, the voltage signal was amplitude modulated with a duty cycle of 22%. Under these conditions, the total power delivered by the generator was 1.2 W. However, as inferred in [20], only two-thirds of it was used to generate the distal plasma jet, while the rest of the power was consumed in the primary DBD reactor. The system was fed with Helium (99.5% purity) at a flow rate of 2.0 slm regulated by a mass flow controller SEC-N100 (Horiba, Osaka, Japan).

For the given parameters, a 1.0-cm-long plasma plume with a power of around 0.8 W can be generated. In the present work, the distance between the nozzle and the treated surface was set to 1.5 cm, while the time of plasma exposure was fixed to 120 s.

### 3.2. Fungal Strain and Growth Conditions

*Candida albicans* SC5314 was the reference strain selected for this study. Fungal strain was cultured on Sabouraud Dextrose (SD) agar. After 24 h of incubation at 37 °C, the fungal cells were suspended in a sterile physiologic solution (NaCl 0.9%) and the cell density was adjusted using a spectrophotometer (λ = 550 nm). The optical density (O.D.) varied accordingly to the cell density needed for each assay: 1.0 × 10^6^ cells mL^−1^ (OD: 0.380), 1.0 × 10^7^ cells mL^−1^ (OD: 0.564)) and 1.0 × 10^8^ cells mL^−1^ (OD: 1.754).

In order to achieve reliable AFM and FTIR analyses of the cells exposed to the different treatments (CAP and caspofungin), *C. albicans* cells were transferred to the surface of standard glass slides.

### 3.3. Antifungal Activity of CAP

Five microliters of the inoculum (10^6^ cells/mL^−1^) were deposited on the surface of a glass slide (1 × 1 cm). After drying for 5 min, another 5.0 μL was added to the same slide. This procedure was repeated until completing 60 μL on each slide. Then, the slide was exposed to the plasma jet for 120 s. The slide was transferred to a tube containing 2 mL of RPMI medium, and the tube was incubated for 24 h at 37 °C. After incubation, the tube was agitated by vortexing and the cell suspension was serially diluted (10^−1^ to 10^−6^). Each dilution was plated on the surface of Sabouraud dextrose agar according to the Miles and Misra method, 1931 [48]. After incubation for 24 h (37 °C), the colonies were counted to determine the CFU/mL^−1^. This assay was performed in triplicate and repeated three times. To determine the CFU for the negative control group, the same steps as described above were repeated. After the drying step, the slides were immediately transferred to the RPMI medium for cell recovery.

**Figure 7:**
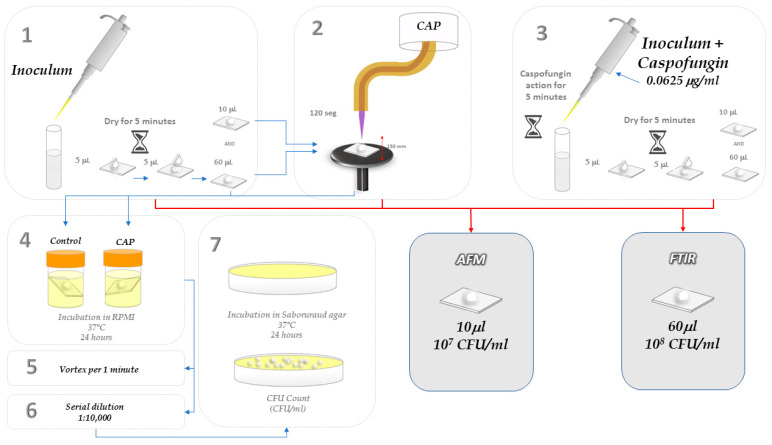
Schematic of the adopted experimental procedure: 1. Preparation of negative control glass slides; 2. CAP treatment; 3. Preparation of glass slides of positive control (Caspofungin); 4. Evaluation of antifungal effects of CAP; 5. Detachment of cells by vertexing; 6. Serial dilutions in 0.9% NaCl saline solution; 7. Plating of dilutions and colony-forming unit (CFU) determination. The glass slides were prepared in the steps 1, 2 and 3 and then analyzed by FTIR and AFM.

The percentage of cell viability reduction was determined by the ratio between the number of viable cells after CAP exposure and the number of cells in the negative control group. The schematic experimental setup is illustrated in Figure 7.

### 3.4. Determination of Minimum Inhibitory Concentration (MIC) of Caspofungin

Caspofungin was used as the positive control due to its proven actions on cell wall synthesis. The minimal inhibitory concentration on *C. albicans* was determined according to the M27-A3 broth microdilution protocol (Clinical and Laboratory Standards Institute, Wayne, PA, USA). Briefly, the test concentrations of caspofungin ranged from 0.0078 μg/mL to 16 μg/mL. Fungal inoculum was prepared and diluted until 1.0 × 10^3^ cells mL^−1^. Then, 100 μL of the diluted inoculum was added to the wells of 96 well-plates containing the different concentrations of caspofungin diluted in RPMI 1640 medium (with glutamine, without bicarbonate, buffered with MOPS to pH 7.0). The plates were incubated for 24 h and the lowest concentration of caspofungin that reduced 50% of fungal growth was considered the MIC.

### 3.5. Morphological and Topographic Analyses of Candida albicans Cells Exposed to CAP by Atomic Force Microscopy (AFM)

Glass slides (1 × 1 cm) containing 10 μL of fungal inoculum exposed to CAP plasma jet were dried for 48 h inside a laminar flow chamber. To evaluate the effect of caspofungin on *C. albicans* cells by AFM and FTIR, 100 μL of caspofungin in the MIC at a concentration of 0.0625 μg/mL was transferred into 100 μL of the inoculum, and after 5 min of caspofungin action, this suspension was dropped into the glass slides. Non-treated samples were used as negative controls. 

The topography maps of the samples were obtained using a Shimadzu (Kyoto, Japan) J3 SPM 9600 atomic force microscope. Measurements were obtained in intermittent-contact mode at a scanning rate of 0.5 Hz. A silicon cantilever (K = 50 N/m) was used. The surface roughness was evaluated by the arithmetic average (Ra) and peak-to-valley height (Rz) values obtained from a 2.81 × 2.81 µm² area using 25 cells from each group. The data analysis was performed using the free software Gwyddion. Version 2.62

### 3.6. Biochemical Analyses of C. albicans Cells Exposed to CAP by Infrared Spectroscopy with Fourier Transform (FT-IR)

Five microliters of the inoculum (10^6^ cells/mL^−1^) were deposited on the surface of a slide glass (1 × 1 cm). After drying (5 min), another 5 μL was added to the same slide. This procedure was repeated until completing 60 μL on each slide. 

The glass slides containing the fungal suspensions, remained overnight in a laminar flow chamber, to eliminate residual water, which could interfere with the FTIR analysis. Data were collected using a Frontier Perkin Elmer 400 spectrometer, Massachusetts, EUA. in the attenuated total reflection (ATR) mode. The scan was performed from 400 to 4000 cm^−1^, at 6 cm^−1^ spectral resolution. A total of 64 scans per sample point were performed according to Adt et al. (2006) [44], thus taking 100 s to obtain an FT-IR spectrum. Nine samples per group were evaluated. The spectra were preprocessed. Initially, the baseline was corrected by the software of the equipment (Opus 4.2, Bruker optics, Ettlingen, Germany), and then the spectra were vector-normalized using Origin Lab 8.0 software. The Savitzky–Golay smoothing filter was applied. A second-order polynomial fit with a window of 11 spectral points was used. 

Subsequently, the calculation of the second derivative was performed to find specific bands, and the deconvolution of bands was performed to calculate band area using the fitting curve resource available in the Origin Lab 8.0 software. The spectra shown in all FTIR figures were an average of nine spectra taken from different samples.

The curves observed in Figure 3a were classified into major biochemical groups, namely polysaccharides (blue), proteins (pink), and lipids (green), as described by [41,42]. The region in which both types of molecules, polysaccharides and proteins, were absorbed was called the mixed region (yellow), as described by [41,42].

### 3.7. Statistical Analyses

The statistical analyses of the data were performed using the Origin Lab 8.0 software. The data were analyzed for normality distribution by the Shapiro–Wilk test. After that, the data were compared by one-way analysis of Variance (ANOVA) and Tukey’s post hoc test. The level of significance was set at 5%.

## 4. Discussion

Previous studies [27,49,50] reported the effect of CAP on the cell viability of *C. albicans*; however, the information about the plasma interaction with the fungal cell walls is limited. In this study, AFM and FTIR were associated to collect information about morphological and biochemical alterations caused by CAP on *C. albicans* cell wall. 

The jet operation parameters selected for this study were based on a previous work of our group, in which the plasma treatment showed an inhibitory effect on *C. albicans* with no cytotoxicity to mammal cells [28,31]. The studies by Handorf et al. [27] showed that the exposure to plasma for 120 s caused damages to the fungal cell integrity. Therefore, considering the objective of this study, we adopted the same period of plasma exposure in order to detect the damages to cell structures, without necessarily causing cell rupture. Moreover, helium was chosen as working gas because it is considered safer for biomedical applications since the discharge current as well as the gas temperature in He are lower than the Argon plasma. 

Cell wall structure changed significantly after exposure to Helium CAP for 120 s. The biochemical profiles after exposure to CAP and caspofungin were similar, with reduction in the presence of β-glucans and increase of chitin. These results suggest the occurrence of a process called unmasking. *C. albicans* cell wall has two layers: an outer one composed mostly by mannan and an inner one, in which the main components are β-(1,3)-glucan and chitin. After unmasking, β-glucans are exposed and the recognition by the host’s immune system is facilitated due to the role of dectin-1, which is a signaling lectin, specific to β-(1,3)-glucan [1] (Hasim et al., 2016).

Another interesting finding was the reduction of the cell size after exposure to CAP, which was also observed after the treatment with caspofungin. These reductions may be associated with a loss of cell content that may occur because of the alterations in the cell wall. The reduction of β-(1,3)-glucan, which comprises 30–60% of the cell wall, can cause an increase in the cell wall permeability and eventually a cell rupture due to osmotic pressure [39].

Substantial changes in the cell’s morphology, such as swelling and ripples deepening, were detected after exposure to CAP and caspofungin. A similar observation was also reported by Quiles et al. (2017) [44] who evaluated the effect of caspofungin (0.06 μg/mL^−1^) on *C. albicans* cells.

The increase in surface roughness may be also related to unmasking process. Similar results were observed in a previous study in *Candida albicans* SC5314 cells treated with caspofungin [1]. After exposure to the drug, the mean quadratic roughness (RMS) of the cells increased significantly compared to the untreated ones. El-Kirat-Chatel et al. (2013) and Formosa et al. (2013) [51] also observed that after exposure to caspofungin, the cell topography was affected with increased roughness.

Although the morphological and topographic changes after exposure to CAP are like those of cells treated with caspofungin [1,44,51,52], the microscopic analysis alone, is not sufficient to confirm that the CAP action on the cell walls is the same as the one of caspofungin. Although AFM generates interesting data, in order to determine a more specific cellular target, the association with other techniques, such as the spectroscopy, i.e., FT-IR, is necessary.

The isolated spectral analysis of the polysaccharides after exposure to CAP revealed a decrease in the bands related to glucans, as described by Quiles et al. (2017) [45], El-Kirat-Chatel et al. (2013) [51] and Hasim et al. (2017) [1]. However, in the study by Quiles et al. (2017), there is no mention of modifications related to mannans. Previous studies [52,53] evaluated the action of caspofungin using other biochemical assays and observed an increase in the expression of mannan and chitin biosynthesis when *C. albicans* is exposed to treatment with caspofungin, similar to the results observed in the present study.

According to Quiles et al. (2017) [44], the increase in chitin synthesis is a mechanism that occurs to preserve cells against mechanical failures caused by the reduction of β-glucans in the cell wall, which favors the action of osmotic pressure. It is believed that the increase in chitin synthesis in cells treated with CAP is also related to the reduction of β-glucans. However, the oxidative stress generated by CAP, predominantly from reactive nitrogen species (RNS) present in the Helium plasma afterglow, can also influence this effect. This phenomenon is triggered in a more expressive form after exposure to CAP when compared to caspofungin and influences the increase in chitin biosynthesis [54,55,56]. According to Pemmaraju et al. (2016) [56], high levels of chitin create a potential for mechanical resistance against the deleterious effects of oxidative stress. By increasing chitin rates, the strength and hardness of the cell wall is increased, thereby maintaining its integrity.

The protein region in the FTIR spectra of the cells exposed to CAP showed a unique behavior, characterized by an increase in the bands’ intensity. For caspofungin, there was a decrease in the intensity of the bands, resulting in bands intensity even lower than the intensity of the bands in the control group. The increase in protein synthesis may be related to the increase in mannan synthesis, as proteins bind and these polysaccharides form the mannoprotein network [1]. The increase in the protein content of CAP-treated samples may also be associated with a phenomenon called moonlighting, which is characterized by a differential location of the protein. Cytoplasmic proteins may be expressed on the cell wall, with a possible protective role against the system’s immune defenses. These proteins are also related to virulence factors [57,58], but the mechanism of secretion of these proteins is still undefined. Some authors [58,59,60] have demonstrated that this phenomenon occurs under conditions of oxidative stress due to the presence of reactive oxygen species (ROS) [54,55,61]. The presence of ROS in the CAP plume, evidenced by the presence of excited atomic oxygen and OH [62], can be associated to the increase in the protein band intensity detected in this study. However, additional analyses should be conducted to confirm this hypothesis.

Chitin polysaccharide bands also absorb in the protein region, and as it could be observed in the second derivative of the spectra, plasma exposure increases the intensity of the chitin peaks in this region. This is an additional factor in the protein emissions in the range from 1500 to 1700 cm^−1^ that can contribute to the general increase in the band’s intensity.

Lipids are the smallest components of the fungal cell wall [57,61]. These lipids are located in the middle of the polysaccharide matrix. The FTIR spectra of CAP-treated cells exhibited a behavior that resembles that of cells treated with caspofungin but was different in relation to the negative control. These results suggest that CAP and caspofungin induced similar effects on lipid structures.

Based on the morphological and biochemical evaluations, it is believed that CAP affects the polysaccharides of *Candida albicans* cell wall, in particular the β-glucans. However, it seems that this is not the only action of the plasma, as an alteration of protein content was also observed. Moreover, the biosynthesis of chitin was more expressive in CAP group than in the caspofungin group.

The studies by Okai et al. (2015) demonstrated that the masking of β-(1,3)-glucan by mannans inhibits the maturation of the macrophage phagosome. According to the obtained results that suggest a possible action on the cell wall glucans, and the known importance of glucans in the fungal cell recognition by the immune system, new questions arise. For instance, whether the structural remodeling was a consequence of direct damage to these cellular structures or if there was some modification at a molecular and intracellular level that influenced the reduction in the synthesis of cellular structures of glucans. It is known that the exposure of *C. albicans* cells to antifungal drugs induced a mechanism of remodeling of the cell wall structure, which influenced the expression of chitin, mannan and glucan genes, altering the cell wall proteome [38,60].

Understanding the detailed mechanism of how the CAP affects the *C. albicans* cell wall will contribute to improving the therapeutical protocols for candidiasis and exploring new associations with conventional drugs with similar targets on the fungal cells. This screening study aimed to contribute to this promising area of knowledge. To deepen these initial observations, further investigations, such as transmission electron microscopy to analyze the morphological damages to cell wall structures and immunofluorescence techniques that allow the detailed study of the structures of the cell wall, should be performed.

## 5. Conclusions

Helium-CAP produced morphological alterations in *C. albicans* cells and affected the cell wall polysaccharide content, causing a reduction of glucans and an increase of mannans and chitins.

## Figures and Tables

**Table 1:** Mean and standard deviation of cell size (μm) and surface roughness (nm) of *Candida albicans* cells exposed to cold atmospheric plasma (CAP) and caspofungin detected by atomic force microscopy.

Groups	Cell Size (μm)		Ra Roughness (nm)		Rz Roughness (nm)	
	Mean	SD	Mean	SD	Mean	SD
CAP	3.41	0.3827	138 *	21	303 *	36
Caspofungin	2.53	0.2998	122 *	11.5	298 *	32
Negative control	3.94	0.1067	99	14	255	29

* *p* < 0.05 in relation to negative control group (ANOVA and Tukey’s post hoc test).

**Table 2:** Mean and standard deviation values of functional group areas in *Candida albicans* cell wall after exposure to cold atmospheric plasma (CAP), caspofungin and negative control.

Functional Groups	CAP		Caspofungin		Negative Control	
	Mean	SD	Mean	SD	Mean	SD
Polysaccharides	0.156 *	0.01034	0.075 *	0.02134	0.209	0.03235
Proteins	0.104 *	0.10358	0.072 *	0.01029	0.099	0.01203
Lipids	0.006 *	0.00617	0.007 *	8.68001 × 10^−4^	0.012	0.00133
Mixed region	0.004 *	0.00429	0.0007 *	6.22024 × 10^−4^	0.002	6.55526 × 10^−4^

* *p* < 0.05 in relation to negative control group (ANOVA and Tukey’s post hoc test).

## Data Availability

The data that support this study are available from the corresponding author upon reasonable request.
